# *Artemisia annua*, a Traditional Plant Brought to Light

**DOI:** 10.3390/ijms21144986

**Published:** 2020-07-15

**Authors:** Axelle Septembre-Malaterre, Mahary Lalarizo Rakoto, Claude Marodon, Yosra Bedoui, Jessica Nakab, Elisabeth Simon, Ludovic Hoarau, Stephane Savriama, Dominique Strasberg, Pascale Guiraud, Jimmy Selambarom, Philippe Gasque

**Affiliations:** 1 Unité de recherche Etudes Pharmaco-Immunologie (EPI), Université de La Réunion, CHU La Réunion site Félix Guyon, Allée des Topazes, CS11021, 97400 Saint Denis de La Réunion, France; pascale.guiraud@univ-reunion.fr (P.G.); jimmy.selambarom@univ-reunion.fr (J.S.); philippe.gasque@gmail.com (P.G.); 2 Faculté de Médecine, Université d’Antananarivo, Campus Universitaire Ambohitsaina, BP 375, Antananarivo 101, Madagascar; mahary11@gmail.com; 3 APLAMEDOM Réunion, 1, rue Emile Hugot, Batiment B, Parc Technologique de Saint Denis, 97490 Sainte Clotilde, La Réunion, France; claude.marodon@wanadoo.fr (C.M.); jess.nak27@gmail.com (J.N.); elisabeth.simon@aplamedom.org (E.S.); lud.hoarau@gmail.com (L.H.); 4 INSERM, UMR 1188 Diabète athérothrombose Thérapies Réunion Océan Indien (DéTROI), Université de La Réunion, 97400 Saint Denis de La Réunion, France; yosra.bedoui.bouhouch@gmail.com; 5 EA929 Archéologie Industrielle, Histoire, Patrimoine/Géographie-Développement Environnement de la Caraïbe (AIHP-GEODE), Université des Antilles, Campus Schoelcher, BP7207, 97275 Schoelcher Cedex Martinique, France; stephane.savriama@gmail.com; 6 Unité Mixte de Recherche Peuplements Végétaux et Bio-agresseurs en Milieu Tropical (PVBMT), Pôle de Protection des Plantes, Université de La Réunion, 7 Chemin de l’IRAT, 97410 Saint-Pierre, La Réunion, France; dominique.strasberg@univ-reunion.fr; 7 Laboratoire d’immunologie clinique et expérimentale de la zone de l’océan indien (LICE-OI) CHU La Réunion site Félix Guyon, Allée des Topazes, CS11021, 97400 Saint Denis de La Réunion, France

**Keywords:** *Artemisia annua*, *Asteraceae*, biological properties, secondary metabolites

## Abstract

Traditional remedies have been used for thousand years for the prevention and treatment of infectious diseases, particularly in developing countries. Of growing interest, the plant *Artemisia annua*, known for its malarial properties, has been studied for its numerous biological activities including metabolic, anti-tumor, anti-microbial and immunomodulatory properties. *Artemisia annua* is very rich in secondary metabolites such as monoterpenes, sesquiterpenes and phenolic compounds, of which the biological properties have been extensively studied. The purpose of this review is to gather and describe the data concerning the main chemical components produced by *Artemisia annua* and to describe the state of the art about the biological activities reported for this plant and its compounds beyond malaria.

## 1. Introduction

The family *Asteraceae* comprises a wide number of genera, of which the genus Artemisia is one of the largest and most widely distributed worldwide [1]. The genus *Artemisia* L. is heterogeneous and consists of over 500 species widely geographically distributed in all continents except Antarctica. The genus acclimatizes to any environment, from sea level to high altitudes [2]. However, most of *Artemisia* species grow preferentially in the Northern Hemisphere and at a lower level in the Southern Hemisphere [3,4]. Species of this genus can be perennial, biennial or annual grasses, shrubs or bushes that are generally aromatic, with erect or ascending stems. The leaves of these plants are alternate, often divided, rarely whole and with smooth edges. The origin of the scientific name of the genus *Artemisia* stems from two major interpretations. The first proposition addresses the name “Artemisia” from the Greek goddess “Artemis” (Diana for the Romans), Zeus’s daughter and Apollo’s sister, who was considered the protector of the wild animals and goddess of the hunt. The second interpretation assigns the origin of the name to the King of Caria’s (Mausolus) sister and wife Artemisia, who was crowned Queen after her husband’s death. The genus Artemisia is commonly known as “wormwood”. Wormwood, though, strictly speaking, refers to Artemisia absinthium L., which is one of the most common and well-known species of the genus [5]. The type species of the genus Artemisia is the *Artemisia vulgaris* [6]. Apart from *Artemisia annua* L., other very well-known species of the genus include *Artemisia absinthium*, *Artemisia abrotanum* and *Artemisia afra*. These species were used to treat fever and malaria, respectively, in China, Europe and Africa [7]. *Artemisia verlotiorum Lamotte* was introduced to, and is still present in, the Mascarene Islands.

**Figure 1:**
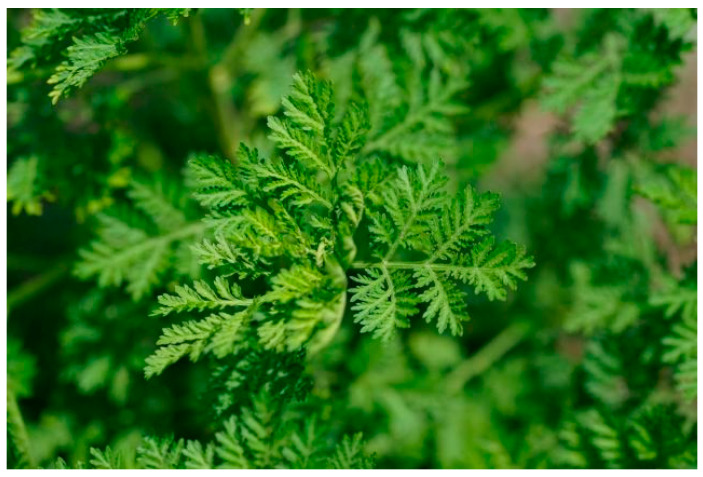
Artemisia annua [9].

*Artemisia annua*, (Figure 1) commonly named as “annual absinthe” is an annual herbaceous herb, hence its name “annua”. The plant is grown in Asia, India, Central and Eastern Europe, in the temperate regions of America, Africa, Australia and in tropical regions [3,7]. It is widely used as a dietary spice, herbal tea and medicinal plant in the mild climates of Asia, such as China and Korea [8].

The literature describes a confusion over the ancient Chinese names of the species *Artemisia annua* L. and *Artemisia apiacea hance*, the latter being native to China, indistinctly referred as “qing hao”. The polymath Shen Gua (1031–1095) of the Song dynasty described two different varieties of qing hao, one with blue–green leaves, and another with yellowish–green leaves in autumn. Based partly on this description, the physician and natural historian Li Shizhen (1518–1593) differentiated between qing hao (blue-green herb) and huang hua hao (yellow blossom herb) in his encyclopedic *Classified Materia Medica* (*Ben cao gang mu*) in 1596 [10]. Currently, *Artemisia apiacea hance* is identified as qing hao, and *Artemisia annua* as huang hua hao [7]. Unlike *Artemisia apiacea hance, Artemisia annua* has been introduced to many other countries in Europe, North America, and the tropics. Seed varieties have been adapted by breeding for lower latitudes, and cultivation has been successfully achieved in many tropical countries, for example in the Congo, India, and Brazil. In contrast. *Artemisia apiacea hance* is less common and is rarely grown outside China [5].

*Artemisia annua* has been used in traditional medicine for many years in Asia and Africa for the treatment of malaria and fever, in the form of tea or pressed juice [11,12]. The current pharmacopoeia of the People’s Republic of China officially lists the dried herb of *Artemisia annua* as a remedy for fever and malaria, at a daily dose of 4.5–9 g of dried herb prepared as an infusion [13]. This is the herbal preparation that has been used for clinical trials.

*Artemisia annua* is also described to have anti-hyperlipidemic, anti-plasmodial, anti-convulsant, anti-inflammatory, anti-microbial, anti-cholesterolemic and antiviral properties [14,15,16]. *Artemisia annua* would also have important pharmacological activities such as anti-inflammatory, antitumor and anti-obesity activities that contribute to the therapeutic effects of the plant [17,18,19].

Several bioactive metabolites have been identified in *Artemisia annua*. The most extensively studied is artemisinin, a lactone sesquiterpene endoperoxide [20]. Due to its antimalarial activity, artemisinin is pivotal in current antimalarial drug strategies [21,22,23]. In addition to this active compound, *Artemisia annua* has also an interesting nutritional profile with the presence of amino acids, vitamins and minerals and essential elements for health [24]. Since its discovery, *Artemisia annua* has been the subject of extensive research on its chemical composition. More than 600 secondary metabolites have been identified throughout the plant [25], including several sesquiterpenoids, triterpenoids, monoterpenoids, steroids, flavonoids, coumarins, alkaloids and benzenoids [26,27,28].

Thanks to this richness, *Artemisia annua* has a large number of other biological properties such as hepatoprotective, antifungal, antitumor, antioxidant, anti-inflammatory and anti-asthmatic activities [8,17,18,27,29,30,31,32,33,34,35].

All these works ascertain the potential of *Artemisia annua* as a candidate for the food, medical, pharmaceutical, cosmetic and nutraceutical industries.

The purpose of this review is to provide a comprehensive synthesis of the various identified chemical compounds of *Artemisia annua* and to describe the state of the art of the biological activities described of this plant and its compounds beyond malaria.

## 2. Chemical Compounds from *Artemisia annua* and Their Biological Activities

Despite enormous geographic diversity, there are almost no morphological differences between the plants of *Artemisia annua*. However, clear differences are observed in the chemical compositions and health benefits of *Artemisia annua* plants according to their geographical location [36,37,38].

The chemical composition and biological properties of the aqueous or alcoholic extracts of *Artemisia annua* can vary considerably depending on its geographical origin, the plant material used and the way it is treated, unlike those of essential oil, which vary only slightly [39].

### 2.1. Monoterpenes

Monoterpenes are a class of terpenes that consist of two isoprene units, with a molecular formula: C_10_H_16_. Monoterpenes may be linear (acyclic) or contain rings. Modified terpenes, such as those containing oxygen functionality or missing a methyl group, are called monoterpenoids. Monoterpenes are secondary metabolites of plants. These molecules, with a very important chemical diversity, allow the plant to defend itself against biotic and abiotic stress factors and act as chemical signals through which the plant communicates with its environment (plants and other organisms) [40].

Monoterpenes are the main components of the essential oil of *Artemisia annua* and give the plant its strong and aromatic fragrance [41]. The main components of the essential oil are 1,8-cineole, α-and-β-pinene, camphene, borneol, camphor, carvone, limonene, α-terpinene and myrtenol [1,39,41,42].

Table 1 describes the structure and biological activities of major monoterpenes of *Artemisia annua* essential oil.

### 2.2. Sesquiterpenes

Sesquiterpenes are a class of terpenes that consist of three isoprene units, with the molecular formula C_15_H_24_. Like monoterpenes, sesquiterpenes may be acyclic or contain rings, including many unique combinations. Biochemical modifications such as oxidation or rearrangement produce the related sesquiterpenoids. Sesquiterpenes have the known role of defense agent (biocide) against organisms outside the plant.

More than thirty sesquiterpenes are present in the *Artemisia annua* plant, mainly located in the aerial parts. The main compounds are artemisinin, arteannuin B and artemisinic acid [83,84,85,86,87]. Table 2 presents the structure and biological activities of major sesquiterpenes of *Artemisia annua*.

The therapeutic value of artemisinin is limited due to its low solubility in both oil and water. Researchers have synthesized a family of artemisinin derivatives including dihydroartemisinin (DHA, active metabolite), artesunate (ART, polar derivative), artemether (lipid-based derivative), arteether (lipid-based derivatives), SM905 (1-(12β-dihydroartemisinoxy)-2-hydroxy-3-tert-butylaminopropane maleate, new water-soluble derivative), artemiside (a 10-alkylamino sulfide derivative, lipophilic with limited water-solubility), artemisone (new 10-alkylamino sulfone derivative with enhanced water-solubility and reduced toxicity) and SM934 (β-aminoarteether maleate, new water-soluble derivative) [91]. Artemisinin and its derivatives can be used in the treatment of various diseases, such as cancer, autoimmune diseases, diabetes, viral infections, parasitosis and atherosclerosis [99]. The antimalarial efficacy of artemisinin is significantly improved when combined with other compounds from *Artemisia annua* such as terpenes, flavonoids, phenolic acids and polysaccharides [100].

## 3. Phenolic Compounds

Phenolic compounds are organic molecules that are widely distributed throughout plants from roots to fruits. These molecules have no direct function in the basic activities of the plant organism, such as growth or reproduction. They are secondary metabolites produced by plants to protect themselves from ultraviolet attacks, but also from animals, often acting as repellents due to their bitterness. Phenolic compounds are molecules with at least one aromatic ring (benzene) bearing one alcohol group, the basic structure called phenol. They are widely present in the plant kingdom in the form of simple (one aromatic ring) or more complex structures (aromatic fused rings), generally of high molecular weight.

Several classes of phenolic compounds are found in *Artemisa annua* aqueous and alcoholic extracts [39,83,101,102,103,104]:

Cyclitol: Quinic acid;

Phenolic acid: Caffeic acid;

Flavonoids: Luteolin, Quercetin, Rutin, Apigenin, Isorhamnetin, Kaempferol, Mearnsetin, Artemetin, Casticin, Chrysosplenetin, Chrysosplenol D, Cirsilineol, Eupatorine.

The *Artemisia annua* antioxidant capacity mentioned in the literature is associated with the high content of flavonoids and the diversity of compound types [24,101].

Table 3 presents the chemical structures and biological activities of major phenolic compounds of *Artemisia annua* extracts.

Artemetin, casticin, chrysosplenetin, chrysosplenol D, cirsilineol and eupatorin are flavonoids from *Artemisia annua* that show some synergic anti-malarial effects with them [7]. Other phenolic compounds increase the antitumor and antimalarial activities of artemisinin [101].

## 4. Coumarins

Coumarins are natural substances derived from benzo-α-pyrone. They have one or more phenolic functions. Coumarins are widely distributed in the plant kingdom. They are formed in the leaves and accumulate especially in the roots and bark, as well as in old or damaged tissues. Coumarins protect the plant from herbivores and pathogenic microorganisms. They are mainly located on the surface and in the organs most exposed to predation (young leaves, fruit and seeds) in order to “save metabolic energy”.

The main coumarins found in *Artemisia annua* alcoholic extracts are scopolin and scopoletin [83]. Table 4 presents the chemical structures and biological activities of major coumarins present in *Artemisia annua* extracts.

## 5. Biological Activities of *Artemisia annua*

### 5.1. Antioxidant Activities

Several studies have demonstrated the antioxidant capacity of *Artemisia annua* [31,39,173], which could be due to the presence of phenolic compounds [30,173].

Messaili et al. [160] demonstrated that the antioxidant activity of *Artemisia annua* was due to the presence of certain families of compounds, namely terpenes, flavonoids and coumarins. It is worth mentioning that a flavonoid named chrysoprenol D (molecular formula C_18_H_16_O_8_) has been identified as the main molecule contributing to the antioxydant activity of this plant. The authors also showed that the total alcoholic extract of the plant had a stronger antioxidant activity than its fractions, demonstrating the synergistic effect of the molecules present in the plant. The compounds of *Artemisia annua* extract were found to act primarily by hydrogen atom transfer rather than single-electron transfer.

Another study showed that the essential oil of *Artemisia annua* had antioxidant properties, based on the use of the 2,2-diphényl-1-picrylhydrazyle (DPPH), 2,2′-azino-bis(3-ethylbenzothiazoline-6-sulfonic acid (ABTS) diammonium salt), Oxygen Radical Absorbance Capacity (ORAC) tests and metal chelating ability using the ferrozine assay [11].

A study investigated the protective effect of the aqueous ethanol extract of *Artemisia annua* against D-galactose-induced oxidative stress in C57BL / 6J mice. The diet containing the extract of *Artemisia annua* reduced serum levels of malondialdehyde and 8-OH-Dg, which are biomarkers for lipid peroxidation and DNA damage, respectively. In addition, feeding the mice with *Artemisia annua* extract diet improved the activity of NQO1 (NAD(P)H: quinone oxidoreductase 1), a typical antioxidant marker enzyme, in organs such as the kidneys, stomach, small intestine and large intestine [18].

### 5.2. Antidiabetic Activities

Aqueous extracts of *Artemisia annua* show significant anti-hyperglycemic and anti-hypoinsulinemia activities in diabetic animals. In fact, significant decrease in blood glucose level occurred in animals receiving 28.5 mg/kg twice a day of the aqueous extract [26]. This may be due to stimulation of the secretion of insulin by β cells, inhibition of α cells of the pancreatic islets, or by enhancing insulin activity [174].

In addition, an important link between oxidative stress, inflammatory response and insulin activity is now well established. This can be explained by the ability of antioxidants to protect against the deleterious effects of hyperglycemia and also to improve glucose metabolism. Generally, these antioxidants are flavonoids which were demonstrated to act on biological targets involved in type 2 diabetes mellitus such as α-glycosidase, glucose cotransporter or aldose reductase [175].

The anti-hypoinsulinemic effect of essential oil components of *Artemisia annua* extract (camphor, germacreneD, artemisia ketone,1,8-cineole) may be attributed to its protective effect against hepatocyte damage through inhibition of the lipopolysaccharide (LPS)-elicited expression of the proinflammatory mediators IL-1β (Interleukin 1 beta), TNF-α (tumor necrosis factor alpha), COX-2 (cyclooxygenase 2) and iNOS (Inducible nitric oxide synthase) [176].

### 5.3. Cytotoxic and Antitumor Effects

Several studies suggest that artemisinin may not be the most active antitumor compound in *Artemisia annua* [94,101]. *Artemisia annua* contains a variety of other biologically active substances [30,101,177], suggesting that this plant could be a source of new herbal anticancer therapies.

Lang et al. [30] demonstrated that *Artemisia annua* extract free of artemisinin has antitumor activity in vitro and in vivo and identified active compounds. In vitro data were validated in two in vivo cancer models, the chick chorioallantoic membrane (CAM) assay and the orthotopic breast cancer xenografts in nude mice. The *Artemisia annua* extract, inhibited the viability of breast (MDA-MB-231 and MCF-7), pancreas (MIA PaCa-2), prostate (PC-3), non-small lung cell (A459) cancer cells. Likewise, the extract’s most abundant ingredients, chrysosplenol D, arteannuin B, and casticin, inhibited the viability of MDA-MB-231 breast cancer cells. The extract induced the accumulation of multinucleated cancer cells within 24 h of treatment and increased the number of cells in the S and G2/M phases of the cell cycle, followed by loss of mitochondrial membrane potential, caspase-3 activation, and the formation of an apoptotic hypodiploid cell population. Further, the extract inhibited cancer cell proliferation, decreased tumor growth, and induced apoptosis in vivo in triple negative breast cancer (TNBC) and MDA-MB-231 xenografts grown on CAM as well as in nude mice.

Essential oil isolated from *Artemisia annua* (100 µg/mL) induced apoptosis in SMMC-7721 hepatocarcinoma cells by nuclear chromatin fragmentation and cytoplasmic condensation [178].

Another study showed that a water-soluble polysaccharide with a molecular weight of 6.3 × 10^4^ Da isolated from *Artemisia annua* inhibited the growth of HepG2 cells in a dose-dependent manner. phenylindole dihydrochloride (DAPI) staining and flow cytometric analysis revealed that the soluble polysaccharide suppressed cell proliferation via induction of the p65-dependent mitochondrial signaling pathway, as evidenced by the increased activation of caspase-3 and -9, negative regulation of Bcl-2 protein, increased regulation of Bax protein and release of cytochrome *c* from mitochondria into the cytosol, and suppression of the nuclear factor κB (NF-κB) p65 [179].

Several mechanisms of action regarding the antitumor activities of artemisinin and its derivatives have been identified [99]. The oxidative stress response has a major role, as it has been demonstrated that the endoperoxide moiety is crucial for the bioactivity of artemisinin-type drugs. Its cleavage leads to Reactive Oxygen Species (ROS) formation and presumably oxidative stress. The authors found numerous statistically significant associations between cellular response to artemisinin and mRNA expression of genes involved in oxidative stress response [180,181,182,183]. Artemisinin induces oxidative DNA-damage in dose-dependent manner [99,148]. ROS and oxidative DNA lesions tremendously affect cellular integrity, leading to perturbations in cellular replication and division mechanisms, which ultimately cause cell cycle arrest and cell death. This mechanism is also true for artemisinin-type drugs. Cell cycle arrest has been reported to occur at G1 or G2 checkpoints, presumably depending on individual defects of tumor cell lines in the cell cycle machinery. All these cascades of events lead to cell apoptosis [99]. Depending on the cell model, both mitochondrial (intrinsic) and the extrinsic FAS-receptor-driven pathways of apoptosis can be induced by artemisinin with upregulated Fas/CD95 expression, breakdown of the mitochondrial membrane potential, cytochrome C release, PARP (poly (ADP-ribose) polymerase) cleavage and caspase 3/9activation [184,185]. Other cell death mechanisms induced by artemisinin-type drugs in tumor cells include non-apoptotic cell death mechanisms such as autophagy, necrosis, necroptosis, oncosis (ischemic cell death), anoikis (anchorage-dependent cell death) and ferroptosis [99]. It has been described that ferrous iron enhances the cytotoxicity of artemisinin-type drugs against tumor cells and that the form of iron-dependent cell death termed ferroptosis is tightly linked to artemisinin and its derivatives [186,187].

### 5.4. Immunomodulatory Effects

Artemisinin and its derivatives have been the subject of several studies on their immunoregulatory properties [188]. They modulate key effectors of the immune system, including toll-like receptors (TLRs) [189,190].

Wojtkowiak-Giera et al. [190] presented two studies demonstrating the immunomodulatory effect of *Artemisia annua* water extracts on TLR2 and TLR4 immune system components. The first evaluated the effects of *Artemisia annua* extracts on the expression of TLR2 and TLR4 in the brains of mice with *Acanthamoeba* infection. The *Artemisia annua* extract significantly reduced the level of TLR2 expression and altered the level of TLR4 expression.

TLRs are a family of transmembrane proteins belonging to several innate immune receptors located primarily on cells of the immune system and others such as lung cells. These receptors play a key role in the recognition of pathogens, including parasites (by recognizing molecular patterns associated with pathogens (PAMPs) or molecular patterns associated with host-derived damage (DAMPs)) and induce inflammatory mediators production [191]. TLR 2 and 4 are the best known and most studied members of this family [192].

The second study evaluated the effects of *Artemisia annua* extracts on TLR2 and TLR4 expression in the lungs of mice with acanthamoebiasis [188]. Extracts from *Artemisia annua* can modulate the expression of both TLRs. The effect of artemisinin and derivatives was suggested to be associated with a decrease in TLR2 expression, TLR4 mRNA expression was found to be increased. *Artemisia annua* extracts were hence suggested to have anti-inflammatory properties by reducing TLR2 mRNA expression.

Similar effects were reported by Li et al. [193] in in vitro experiments where artesunate, a widely used artemisinin derivative, inhibited the secretion of TNF-α from murine peritoneal macrophages induced by heat-killed *Staphylococcus aureus* via decreased TLR2 mRNA expression.

Artesunate also decreased the expression of TLR4 and TLR9 mRNA. TLR4 is a receptor that induces inflammatory response activation by the recruitment of adaptor proteins such as MyD88 that leads to the activation of the nuclear factor NF-κB and the production of pro-inflammatory cytokines. It should be noted that artesunate can inhibit the LPS-induced expression of TLR4, MyD88 and NF-κB by blocking the degradation of the inhibitor of NF-κB (IκB) [194].

Artemisinin and its derivatives have been tested for their anti-inflammatory activities in numerous models of auto-immune and allergic conditions.

Artesunate, dihydroartemisinin, artemether and the water-soluble derivative SM905 have been reported to possess protective effects against experimental models of rheumatoid arthritis (RA) [195,196,197,198].

In an experimental RA model, the attenuation of inflammatory symptoms and prevention of tissue damage were obtained with artesunate. Artesunate was found to induce the suppression of proinflammatory cytokines including TNF-α, GM-CSF, IL-1β, IL-6, IL-8 and IL-17α via inhibition of the mitogen activated protein kinase (MAPK), phosphoinositide 3-kinase (PI3K)/Akt and NF-κB signaling pathways [196,198,199]. Artemisinin and derivatives have also been shown to exert anti-angiogenic activities in RA, acting as inhibitors of angiogenesis-related factors such as matrix metalloproteinase-2 (MMP-2) and MMP-9, Vascular Endothelial Growth Factor (VEGF) and hypoxia-inducible factor-1α (HIF-1α) [196,200]. Artemisinin and its derivatives have demonstrated effective antiarthritic properties in RA, with comparable efficacy but a significantly reduced side effect profile as compared to methotrexate [17].

In an experimental murine model of Systemic Lupus Erythematosus (SLE), oral artesunate at 125 mg/kg/d over 16 weeks exhibited comparable immunosuppressive effects to cyclophosphamide, by repressing monocyte chemoattractant protein 1 (MCP-1) and B-cell-activating factor (BAFF) levels, leading to a significant reduction in anti-nuclear antibody and anti-double-strand DNA (dsDNA) antibody production, proteinuria, serum creatinine as well as related renal pathology [201].

Studies have also revealed that a 3–8 week regime of oral water-soluble artemisinin analog SM934 (2.5 and 10 mg/kg/d) exhibited pronounced suppression of proteinuria, glomerulonephritis, development of Th-1 and Th-17 cytokine profiles, and increases in anti-dsDNA, IgG2a and IgG3 antibodies, while promoting increases in Th-2 responses, and serum IL-10 and IL-4 levels in experimental murine models of SLE [202,203].

SM934 demonstrated mixed actions on different subsets of T cells, suppressing the memory/effector T cells, while promoting regulatory T cell development. Notably, these studies have revealed that SM934 can exhibit extensive protective effects in chronic systemic inflammatory condition, comparable to a clinically effective corticosteroid drug like prednisolone [202] or immunosuppressant like rapamycin [203].

In lupus nephritis, a severe and frequently-occurring secondary kidney-specific inflammation following SLE, oral dihydroartemisinin (5–125 mg/kg/d) was found to suppress serum levels of anti-dsDNA antibody and TNF-α and abrogate renal pathology in mice via blockade of NF-κB p65 subunit nuclear translocation [204]. Besides, oral artesunate (150 mg/kg/d) has demonstrated stronger protective effects than prednisone in experimental lupus nephritis, by lowering serum levels of TNF-α and IL-6, and NF-κB p65 subunit and transforming growth factor beta 1 (TGF-1β) expressions in renal tissues [205]. Furthermore, artesunate combined with prednisone was found to induce higher expression of glucocorticoid receptor α (GRα) in peripheral blood mononuclear cells (PBMCs) and to enhance transcriptional coactivator P300/CBP protein expression in renal tissues when compared to prednisone alone in lupus nephritis mice [206]. 

Artesunate has been shown to possess therapeutic actions against inflammatory bowel disease (IBD) [207]. Artesunate (150 mg/kg/d) dramatically mitigated colon pathology and inflammatory damage in experimental colitis induced by dextran sulfate sodium salt (DSS) or trinitrobenzene sulfonic acid (TNBS). These anti-inflammatory effects of artesunate corroborated well with the suppression of Th-1 and Th-17 cytokines, IFN-γ and TNF-α via the inhibition of NF-κB activities [17].

Artemisinin and its derivatives have been also shown to have anti-allergic activities, which are linked to their immunosuppressive effects mediated by the downregulation of NF-κ p65 subunit, T-bet and IFN-γ expressions [208]. A Chinese trial on 90 subjects with allergic skin disorders has demonstrated that topical artesunate exerts potent efficacy against eczema, erythema multiforme, polymorphous sunlight eruption and hydroa aestivale, and moderate effectiveness against atopic dermatitis, psoriasis vulgaris and dermatomyositis [17].

Cheng et al. [209] demonstrated that artesunate (3–30 mg/kg) prevented IgE-mediated vascular permeability in a passive cutaneous anaphylaxis mouse model, and blocked IgE-induced mast cell degranulation in the lungs, increase in plasma histamine level, and subsequent hypothermia. In RBL-2H3 cells and mature human mast cells, artesunate was found to directly inhibit IgE-induced mast cell degranulation, by blocking Syk tyrosine kinase phosphorylation, the downstream phospholipase Cγ (PLCγ) activation, and elevation in inositol trisphosphate (IP3) and intracellular Ca^2+^ levels. These findings strongly support a therapeutic role for artemisinin and its derivatives in the treatment of mast-cell-mediated allergic responses.

Artesunate has been shown to protect against experimental allergic asthma. At 3–30 mg/kg/d, artesunate given intraperitoneally markedly inhibited both ovalbumin- and house dust-mite-induced total and eosinophil counts in bronchoalveolar lavage fluid, anti-inflammatory effects comparable to dexamethasone [210,211]. Furthermore, artesunate drastically suppressed aeroallergen induced increases in Th-2 cytokines and chemokines, IL-17, IL-33, MUC5AC, and adhesion molecules in the airways [210]. These protective effects by artesunate in allergic asthma have been associated with its pronounced inhibition of the PI3K/Akt signaling cascade and NF-κB activation. In contrast to the formation of free ROS via cleavage of the endoperoxide bond by heme iron in its structure as a mechanism to kill *Plasmodium* spp. parasites and to induce cytotoxic effects in cancer cells, in allergic asthma, artesunate was found to decrease the levels of oxidative and nitrosative damage markers including 8-hydroxy-2-deoxyguanosine, 8-isoprostane and 3-nitrotyrosine, in inflamed airways. These antioxidative effects of artesunate were correlated with the inhibition of expression of NADPH oxidases and iNOS, and elevation of superoxide dismutases and catalase, probably via the induction of nuclear factor (erythroid-derived 2)-like 2 (Nrf-2) by artesunate in allergic airways [210]. Four cases of IgE-mediated anaphylactic reactions to oral and intravenous artesunate have been described. These allergic reactions to artemisinin and its derivatives are considerably rare [212].

Artemisinin was demonstrated to be capable of extenuating amyloidogenesis and neuroinflammation in a model of Alzheimer’s disease (AD) in APPswe/PS1dE9 double transgenic mice [213]. Artemisinin (40 mg/kg) given intraperitoneally daily for 30 days abrogated β-secretase activity and decreased neurotic plaque burden in AD mouse model. These anti-inflammatory effects of artesunate have been ascribed to the inhibition of NF-κB activity and the activation of NALP3 inflammasome. Another therapeutic prospect for artemisinin and derivatives has been investigated in an experimental rat model of endometriosis. Artesunate at 150 and 300 mg/kg/d, given intragastrically for 4 weeks, increased apoptosis index and significantly reduced Bcl-2 and microvascular density of the implanted ectopic endometrium, with protective effects comparable to a modified progestogen danazol [214].

### 5.5. Antibacterial and Antifungal Activities

The essential oil of *Artemisia annua* has been the subject of numerous studies to test its antibacterial and antifungal activities. Tests were carried out both on the whole oil and on its principal components such as camphor, 1,8-cineol, α-pinene, and artemisia ketone [215]. 

The main gram-positive bacteria tested with *Artemisia annua* volatiles organic components obtained by hydrodistillation were *Staphylococcus aureus* [11,216,217,218,219,220], *Enterococcus hirae* [217], *Enterococcus faecalis* [219], *Streptococcus pneumonia, Micrococcus luteus* [220], *Bacillus cereus* [219], *Sarcina lutea* [11], *Bacillus subtilis* [220], *Bacillus* spp. [217], and *Listeria innocua* [221]. For the gram-negative bacteria, *Escherichia coli* [217,218,219,220], *Escherichia coli* UPEC-Uropathogenic [220], *Escherichia coli* ETEC-Enterotoxigenic, *Escherichia coli* EPEC-Enteropathogenic, *Escherichia coli* EIEC Enteroinvasive, *Escherichia coli* STEC-Shiga-toxin producer [222], *Shigella* sp., *Salmonella enteritidis, Klebsiella pneumoniae* [11], *Haemophilus influenzae* [220], and *Pseudomonas aeruginosa* [218,219,220] were tested. All microorganisms tested were inhibited by this essential oil [11].

The main gram-positive bacteria tested with methanol, chloroform, ethanol, hexane, and petroleum ether extracts of *Artemisia annua* were *Staphylococcus aureus* [13,219], *Enterococcus faecalis* [219], *Micrococcus luteus* [13], *Bacillus cereus* [13,219], *Bacillus subtilis, Bacillus pumilus* [13], and *Bacillus* sp. [219]. For the gram-negative bacteria, *Escherichia coli* [13,219], *Escherichia coli* UPEC [219], *Salmonella typhi* and *Pseudomonas aeruginosa* [13,219] were tested. All microorganisms tested were inhibited.

Li et al. [27] studied the antifungal activity of *Artemisia annua* extracts against *Fusarium oxysporum*, *Fusarium solani* and *Cylindrocarpon destrutans* which are three fairly common agricultural fungal pathogens causing root rot disease in the cultivation of plants such as medicinal materials and crops. This study revealed that a coumarin derivative present in the plant *Artemisia annua* with an additional acetyl group attached to C-6 has a wide range of antifungal properties and is active against the three agricultural pathogenic fungi.

Further studies have been performed with the main components present in *Artemisia. annua* essential oil. These studies showed that artemisia ketone is the oil component that has the greatest antimicrobial activity. In fact, it turned out to be effective against bacteria and some fungi (*C. albicans* and *A. fumigatus*) at very low concentrations (range 0.07–10 mg/mL). The other compounds tested in the studies have produced variable results. However, it should be emphasized that all the compounds tested were active (range 1.25–5 mg/mL) against *A. fumigatus*, a dangerous microorganism frequently responsible for nosocomial infections in immunocompromised individuals [215].

The antifungal activity of the essential oil was also evaluated against economically important foliar and soilborne fungal pathogens of tomato. The essential oil was active against *Sclerotinia sclerotiorum*, *Botrytis cinerea*, *Phytophthora infestans*, and *Verticillim dahliae* [223].

### 5.6. Antiviral Activities

There is evidence of in vitro and in vivo antiviral activities of the *Artemisia annua* extract as well as artemisinin and its derivatives. Tested viruses include both DNA and RNA viruses [224].

A methanolic extract of *Artemisia annua* was tested in a syncytium inhibition assay, which is based on the interaction between the HIV-1 envelope and the CD4 cell membrane protein on T-lymphocytes [225].

An in vitro study has shown that tea infusion of *Artemisia annua* has anti-HIV activity. The presence of HIV-malaria coinfection in malaria endemic areas has raised the question of anti-HIV activity of *Artemesia annua* and *Artemisia afra* folksy used for malaria treatment. It has rapidly been demonstrated that artemisinin was not the main compound by which these plants exerted their anti-HIV activities. In vitro, *Artemisia annua* tea infusion was found to be highly active, with IC50 values as low as 2.0 μg/mL, while artemisinin was inactive at 25 µg/mL [16]. Nevertheless, in vitro models of NL3.4 HIV-1 infected PBMCs showed that 10 µM of artemisinin inhibited HIV-1 replication by 60% [226]. Jana et al. [227] found that three of their six synthesized 1,5-disubstituted 1,2,3-triazole dihydroartemisinin derivatives showed significant anti-HIV activity with IC50 values ranging from 1.34 to 2.65 µM, while Effert et al. [228] only found weak inhibition rates against two HIV-1 strains.

Artemisinin and its derivatives from the plant *Artemisia annua*, in particular artesunate, have potent inhibitory effects against double-stranded DNA viruses, including CytoMegaloVirus (CMV), Herpes Simplex Virus 1 (HSV-1), Human Herpes Virus 6A (HHC-6A) and Epstein–Barr Virus (EBV) [229,230].

Most of the studies on the antiviral effects of artemesinin and its derivatives concern the human cytomegalovirus (HCMV) [224]. Effert et al. [228] demonstrated that the artemisinin derivative artesunate inhibited the replication of both sensitive and ganciclovir-resistant strains of HCMV. The authors found that artesunate both inhibits the viral activation of the cellular transcription factor NF-κB and Sp-1 and downregulates the phosphorylation of the upstream kinase P13K. The anti-HCMV activity of artemisinin derivatives was confirmed by other authors, using artemisinin-based dimer and trimer molecules and trioxane-ferrocene hybrids. All the derivatives were highly active and showed very low IC50 value compared to artesunate, artemether, dihydroartemisinin or ganciclovir [97,231,232,233,234]. Hutterer et al. [234] described the drug to bind to NF-κB RelA/p65, this way inhibiting the cell HCMV-upregulated NF-κB activity. The same author described the anti-CMV activity not only for HCMV strains but also for rat, guinea pig and mouse CMV strains. 

The use of combined therapy strategies to fight against ganciclovir-resistant HCMV also brought to light the importance of anti-HCMV activity of artemisinin and its derivatives. Such combinations were found in patients with complications of CMV infection after hematopoietic stem cell transplantation. Oral administration of artesunate isolated from *Artemisia annua* (100 mg/d) resulted in a rapid reduction in viral load (1.7 to 2.1 log reduction) in whole blood and improved hematopoiesis within 10 days [235].

Activities of artesunate were also evaluated with Human Herpes Simplex Virus 1 (HSV1). Effert et al. [228] showed that artesunate strongly inhibited HSV1 in vitro, with no effect on cell viability. In the same vein, Canivet et al. [230] showed an improved outcome of HSV1-induced encephalitis in mice treated with combination of artesunate-valacyclovir compared to valacyclovir monotherapy. The authors also described a decrease in proinflammatory cytokines (IL-1β, IL-2, IL-6, IFN-γ) and chemokines (CCL2, CCL4, CCL6) with the combined treatment compared to valacyclovir alone.

Data on the activities of artemisinin and its derivatives on Human Herpes Virus 6 (HHV6) are inconclusive. Some authors described an inhibitory effect of artemisinin on HHV-6A replication and early and late protein synthesis with an IC50 of 3.80 ± 1.06 μM with no drug-induced apoptosis or necrotic cytotoxicity [230], while such an observation was not found in another study on HHV-6A and HHV-B [236]. Hakacova and al. [237] described a case of a decreased HHV-6B DNA in endomyocardial biopsies of a child with HHV-6 myocarditis and an improvement in clinical status after artesunate treatment, with no adverse effects of the molecule.

Artesunate has also been shown to be effective against hepatitis B virus (HBV) replication [238]. Artesunate was found to suppress HBV surface antigen (HBsAg) secretion with an IC50 value of 2.3 μM, and to reduce HBV-DNA levels with an IC50 value of 0.5 μM [239] in vitro, at a concentration range below the plasma drug concentration required in anti-malarial treatment (~ 7 μM) [240].

Other studies with DNA viruses showed that artesunate inhibited the polyomavirus BK (BKV) infection in human primary proximal tubular epithelial cells. The loads of extracellular BKV DNA, reflecting viral progeny production, were reduced in a concentration-dependent manner. At 10 μM, artesunate reduced the extracellular viral load by 65% and early large T antigen mRNA and protein expression by 30% and 75%, respectively. It also decreased DNA replication by 73%; and late VP1 mRNA and protein expression by 47% and 64%, respectively. The authors also highlighted that artesunate was inhibiting the proliferation of the proximal tubular epithelial cells in a concentration-dependent manner. The inhibition mechanism involved was a cytostatic rather than a cytotoxic mechanism [241]. The same authors described the inhibitory effects of artesunate on the human JC polyomavirus (JCPyV) in a concentration-dependent manner. With an EC50 of 2.9 µM, artesunate decreased extracellular viral DNA load correlated with a decreased expression of capsid protein VP1 and a reduced release of viral progeny. Cell cytotoxicity was observed for high concentrations of artesunate [242].

In human papillomavirus (HPV)-immortalized and transformed cervix cells, apoptosis was observed with artesunate and dihydroartemisinin treatment. Cell death induced by dihydroartemisinin involved the activation of the mitochondrial caspase pathway and was independent of the p53 pathway. However, no change was found regarding the HPV-related oncogene expression. These observations lead the authors to the conclusion that dihydroartemisinin might be useful for the topic treatment of mucosal HPV lesions, including lesions which had reached the neoplastic state [243]. The same antiproliferative effect was described using artemisinin in HPV39-infected cervical carcinoma cells [244].

Regarding RNA viruses, it has been demonstrated in vitro that artemisinin and its derivatives are also effective against single-stranded RNA viruses from the *Flaviviridae* family including Hepatitis C Virus (HCV) [245,246,247] and bovine viral diarrhea virus (BVDV) [248,249]. Co-treatment with hemin or ferrosanol resulted in enhanced anti-*Flaviviridae* activity of artemisinin derivatives (224).

In a case report about a malaria patient suffering from dengue shock syndrome acute renal failure, a treatment with intravenous artemisinin in addition to standard therapy was successful. However, the specific effect of artemisinin against the dengue virus (DENV) has not been demonstrated [250]. Artesunate seems not to be active against influenza viruses [228].

### 5.7. Antiparasitic Activities

#### 5.7.1. Antiplasmodial Activity

Malaria patients in Central Africa treated with *Artemisia annua* tea, at a dose corresponding to the recommendations of the Chinese pharmacopoeia, have shown a very rapid disappearance of malaria parasites in the blood.

Five malaria patients treated with *Artemisia annua* tea showed a rapid disappearance of the parasitemia in 2 to 4 days. An additional trial with 48 malaria patients showed a disappearance of parasitemia in 44 patients (92%) in 4 days. Both trials showed a clear improvement in symptoms [12].

A double blind, randomized clinical trial with 957 malaria-infected patients had two treatment arms: 472 patients for artesunate-amodiaquine and 471 for *Artemisia* extracts (248 *Artemisia annua*, 223 *Artemisia afra*). Artesunate-amodiaquine-treated patients were treated per manufacturer posology, and Artemisia-treated patients received 1 L/d of dry leaf/twig infusions for 7 days; both arms had 28 days follow-up. Trophozoites disappeared after 24 h with *Artemisia annua* treatment but took up to 14 days to disappear in patients treated with artesunate-amodiaquine. Cure rates for Day 28 defined as the absence of parasitaemia were 91%, 100% and 30% for adults for *Artemisia afra, Artemisia annua* and artesunate-amodiaquine, respectively. The onset of fever took 48 h for artesunate-amodiaquine, but 24 h for *Artemisia annua* [251]. 

A specially prepared ether extract of *Artemisia annua*, when fed to mice infected with malaria, was effective in 95–100% of cases [252]. However, artemisinin did not kill the liver stages of the parasite, was metabolized rapidly, and remained in the bloodstream for only a few hours [253]. Hence, it cannot be used as a prophylactic drug. The World Health Organization now recommends the use of artemisinin combination therapies for the first-line treatment of uncomplicated malaria [254], to reduce the risk of parasite resistance and recrudescence. 

#### 5.7.2. Anti-Helminthic Activities

Schistosomiasis affects 250 million people in 78 countries with 280,000 deaths each year [255] and with an estimated 172 million infected in sub-Saharan Africa [256]. The disease is caused by trematode worms of the genus *Schistosoma* with six species (*S. haematobium, S. mansoni, S. japonicum, S. mekongi, S. intercalatum, and S. malayensis*). *S. mansoni* and *S. haematobium* are the main responsible agents for intestinal and urinary schistosomiasis, respectively, that occur mainly in Africa (230). The majority of in vivo investigations reported an anti-schistosomal effect of artemether. Other derivatives (artesunate, arteether, dihydroartemisinin) were studied to a lesser extent. The drugs have been applied to animals *per os* or intragastrically. Artemisinin derivatives strongly reduced the total worm rates independent of application routes, with observed stronger reduction rates for female than male worms. Artemisinin derivatives also significantly reduced worm eggs burden and egg-caused granulomata in the liver of host animals. It has been described that artemether and arteether were as active against praziquantel-resistant *S. japonicum* as they were against the sensitive strain [33]. As it has been proposed that cleavage of the endoperoxide moiety, which is crucial for bioactivity, is facilitated in the presence of ferrous iron (Fe^3+^) from hemin or other sources by a Fenton-type reaction [21,22,23,24], and the combination of artemether and hemin led to higher worm reduction rates than artemether alone [35]. Artemisinin and its derivatives were found to exert oxidative stress in the worms, which leads to glutathione (GSH) depletion and lipid peroxidation. Several anti-oxidant enzymes were reported to be able to inhibit, e.g., glutathione S-transferase (GST), glutathione peroxidase (GPx), glutathione reductase (GR), superoxide dismutase (SOD), cytochrome C peroxidase (cytC). The impaired oxidative stress response in the parasite led to severe morphological damage, e.g., lesions in tegument (swelling, vesiculation, erosion, and peeling), subtegumental musculature, parenchymal tissue, gastrodermis and degeneration of male and female reproduction organs. Furthermore, a number of metabolic enzymes were inhibited, such as glucose-6-phosphate isomerase (GPI), phosphofructokinase (PFK), glyceraldehyde-3-phosphate dehydrogenase (GAPDH), lactate dehydrogenase (LDH), phosphoglycerate kinase PGK), pyruvate kinase (PK), phosphoglycerate mutase (PGAM), aldolase (ALDO), enolase (ENO), hexokinase (HK), malate dehydrogenase (MDH), malic enzyme (ME), glucose-6-phosphate dehydrogenase (G6PD), 6-phosphogluconate dehydrogenase (PGD), mannose-6-phosphateisomerase (MPI), alkaline and acidic phosphatases (ALP, ACP), adenosine triphosphatase, Ca^2+^-ATPase, Mg^2+^-ATPase, and Na^+^/K^+^-ATPase. Both oxidative and metabolic stress led to damage to tegument, tissues and reproductive organs, as well as growth accompanied by decreased worm length [257].

Artemisinin and its derivatives were also found to be active against a number of nematodes. In an in vitro study, artemether treatment caused cuticular changes on adult *Toxocara canis* similar to those induced by albendazole sulfoxide with faster onset of action [258]. Several potential mechanisms of action were described, including disruption of the functions of ion pumps on the apical plasma membrane, of sarcoplasmic/endoplasmic reticulum Ca^2+^-ATPase PfATP6, or mitochondria of the parasite [258,259]. Artemisinin has also been reported to treat adult and larva *Toxocara spiralis* in both in vitro and in vivo studies). Incubation of adult *Toxocara spiralis* worms in 0.05 mg/mL artemisinin solution for 24 h revealed marked swellings, sloughing, and blebbing of the cuticle and loss of normal creases, ridges, and annulations of fissures and vesicles [260]. Abou Rayia et al. [260] observed a significant decrease in the mean number of adult worms in the small intestine and the total larvae in muscles of mice, 75% (17.6 ± 1.82) and 72% (12,490 ± 336.15), respectively, after treatment with an oral dose of 400 mg/kg of artemisinin. The results were comparable to those obtained with mice treated with mebendazole showing a 78.7% (15 ± 1.41) reduction in adult worm number and a 68% (14,300 ± 877.5) reduction in muscle larva number, respectively. Histopathology of the small intestine showed a reduction in the inflammatory infiltrates with both drugs and a significant reduction in both the number of deposited larvae and the intensity of the inflammatory infiltrate in the skeletal muscle of the mice. The reduction in the number of deposited larvae was more evident in the artemisinin-treated group. Moreover, the extent of reduction in COX-2 and VEGF expressions in the cytoplasm of inflammatory cells was greater in mice treated with artemisinin than in those treated with mebendazole. Mechanisms of action of artemisinin towards adult and larva worms of *Toxocara spiralis* are assumed to derive from damage by toxic free radicals and the inhibition of the parasite angiogenesis by suppression of VEGF expression, which may affect the nutrition status and waste removal of the larva worms [261].

Other nematodes on which artemisinin and *Artemisia* extracts have been tested include *Haemonchus contortus* in ruminants and plant nematodes (*Meloidogyne* spp., *Globodera rostochiensis*, and *Xiphinema index*) [262].

Many other helminthic infections were tested with artemisinin and its derivatives including cestode infections (*Echinococcus* spp., *Taenia crassiceps*) and other trematodes infections (*Echinostoma* spp., *Fasciola* spp., *Clonorchis sinensis, Opisthorchis viverrini, Paragonimus westermani, Heterophyes heterophyes*, and *Paramphistomum microbothrium*) [263].

#### 5.7.3. Activity against Other Protozoa

Beside the outstanding antimalarial and antischistosomal activities, artemisinin and its derivatives also possess activities against other protozoan parasites. 

Many studies describe the in vitro and in vivo activities of artemisinin and its derivatives against protozoan parasites, including Leishmania spp., Trypanosoma spp., Toxoplasma gondii, Neospora caninum, Eimeria tenella, Acanthamoeba castellanii, Naegleria fowleri, Cryptosporidium parvum, Giardia lamblia, and Babesia spp [264].

A study on the activity of *Artemisia annua* on the genus Acanthamoeba showed that water, alcohol and chloroform extracts from *Artemisia annua* can be applied in general and local treatment or in combined therapy with antibiotics in the treatment of acanthamoebiasis. Extracts from *Artemisia annua* showed not only in vitro but also in vivo effects. The pure artemisinin preparation affected amoebae from 100 to 300 times more strongly than the studied extracts. The most active anti-amoeba extract was chloroform extract. Studies carried out on experimental animals infected with amoebae showed that the application of these extracts significantly prolonged the survival of the animals [265].

## 6. Conclusions

*Artemisia annua* is widely distributed throughout the world. It is one of the most important plants used in traditional medicine in China and Africa. It has been brought to light again very recently in Madagascar for the prevention and treatment of COVID-19. The aim of this review was to list the molecules most present in this species, regardless of its geolocation, and to describe what is currently known about their properties in the treatment of various diseases in vitro and in vivo models. On the whole, the main objective of this review was to bring together all the available scientific research that has been conducted on this species.

Differences in the chemical composition of *Artemisia annua* extracts can be linked to several factors: geolocation, plant parts used, methods of sample preparation and extraction, genetic differences and cultivation process. These variations can influence the biological properties of this plant. Future research should focus on establishing a reproducible quality control protocol to minimize metabolic variations between individuals. This quality control should include the climate, the season of harvest, the humidity, the richness of the soil, the altitude, the maturity of the plant and the part of the plant used.

*Artemisia annua* has been extensively investigated and shows promising activities: antiplasmodial, antiviral, antimicrobial, antitumor, antiinflammatory, antioxidant. Studies present artemisinin as the active compound of this plant especially for the antimalarial activity. However, other studies show that the species *Artemisia afra*, which does not produce artemisinin, has similar antimalarial properties to *Artemisia annua*. The fact that artemisinin is absent would be beneficial in the fight against parasite resistance. Bio-guided studies should be considered to identify the active molecule(s). A comparative study of the compounds present in the two species shall be considered and in vitro and in vivo experiments shall be considered. In Madagascar, a drink based on an infusion of *Artemisia annua* in mixtures with other plants to prevent and combat COVID-19, is recommended; however, in vitro and in vivo studies are needed to validate these claims. In addition, this plant is not authorized in the French Pharmacopoeia. To date, the State of Madagascar has not received any marketing authorization for this drink. Studies to check for toxic or harmful effects of this plant are extremely important and necessary. 

On the basis of the data presented in this review, it can be concluded that *Artemisia annua* may become a future flagship species in the treatment of various pathologies, if the problems associated with quality control and authorization for use could be resolved and, more importantly, if we can identify the potentially active component(s), in particular the secondary metabolites active against the new coronavirus SARS-CoV-2 responsible for COVID-19. Studies are in progress in this direction but also on the comparison between *Artemisia annua* and *Artemisia afra* in our laboratory. Artemisinin is a compound of interest which is not necessarily the most active molecule of the genus *Artemisia* and does not have the most promising antiviral activity. With this in mind, a clinical trial began in early July in Madagascar to test the antiviral effect of artesunate, a derivative of artemisinin coupled with a high dose of vitamin C, against SARS-CoV-2. The first results will be known by August. The use of artesunate and not artemisinin shows that it is not obvious that this molecule has the most interesting bioactivity.

## Figures and Tables

**Table 1:** Structure and biological activities of major monoterpenes of *Artemisia annua* essential oil.

Molecule	Structure	Activities	Ref.
1,8-cineole	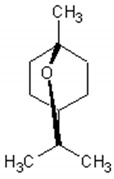	Insecticidal, expectorant, anti-inflammatory, antibacterial, antifungal, antitumor	[43,44,45,46,47,48,49]
α-and-β-pinene	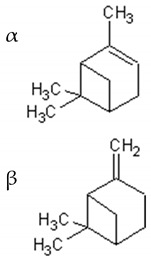	Antimicrobial, anti-hypertensive, antinociceptive, anti-inflammatory, flavor and fragrance purpose, food additive	[50,51,52,53]
Camphene	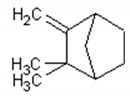	Insecticidal, antitumor, anti-inflammatory, antifungal, antigastriculcer	[54,55]
Borneol	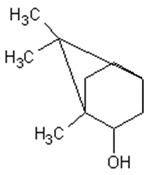	Analgesia, anti-inflammatory, anesthesia, neuroprotective	[56,57,58,59,60]
Camphor	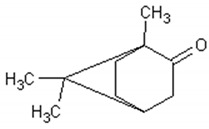	Anti-implantation, antiestrogenic, anticonvulsant, antitussive, uterotrophic, nicotinic receptor blocking, estrogenic, attractant, fragrance purpose, food additive	[61,62,63,64,65,66,67,68,69,70]
Carvone	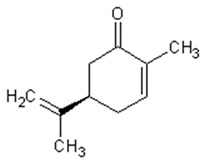	Anti-inflammatory, anti-hyperlipidemic, anti-microbial, anti-carcinogenic, chemopreventive, anti-hypertensive, immunomodulator	[71,72,73,74]
Limonene	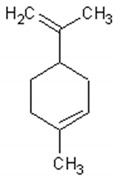	Antioxidant, antigenotoxic inhibition of angiogenesis, antitumor	[75,76,77]
α-terpinene	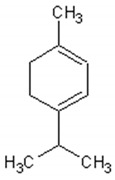	Antioxidant	[78]
Myrtenol	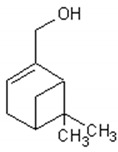	Analgesic, anti-inflammatory, antioxidant, mutagenic, antiaging, neuroprotective, anti-diabetic, antitumor, protects against LDL (Low Density Lipoprotein) oxidation and lung diseases.	[79,80,81,82]

**Table 2:** Structure and biological activities of major sesquiterpenes of *Artemisia annua*.

Molecule	Structure	Activities	Ref.
Artemisinin	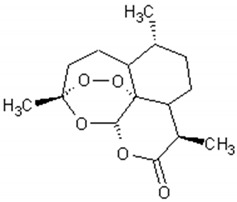	Antiviral, antitumor, antimalarial, antiparasitic, anti-inflammatory, antifibrotic	[88,89,90,91,92]
Arteannuin B	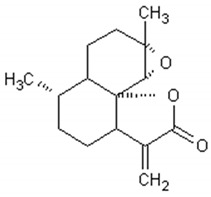	Antiviral, antitumor, anti-inflammatory, larvicidal	[93,94,95,96,97]
Artemisinic acid	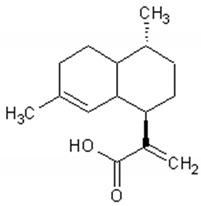	Regulator of adipocyte differentiation	[98]

**Table 3:** Structure and chemical activities of major phenolic compounds present in *Artemisia annua*.

Molecule	Structure	Activities	Ref.
Quinic acid	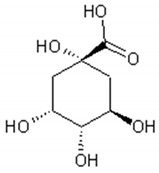	Antioxidant, lipolytic, antiobesity, inhibitor of hepatic glucose-6-phosphate translocase, antiviral	[105,106,107,108]
Caffeic acid	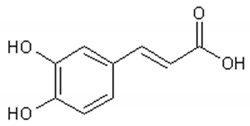	Antiviral, antimicrobial, antiinflammatory, antitumor, antiAlzheimer, anti-diabetic, cardiovascular protector	[109,110,111,112,113,114,115,116]
Luteolin	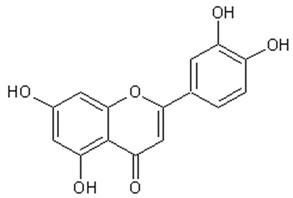	Antitumor, antioxidant, antiinflammatory, nervous system protector, cardiovascular protector	[117,118,119,120,121,122]
Quercetin	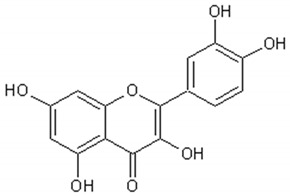	Antioxidant, vasodilator effect, antiinflammatory antitumor, cardiovascular protector, neurodegenerative diseases protector, antiviral	[123,124,125,126,127,128,129,130]
Rutin	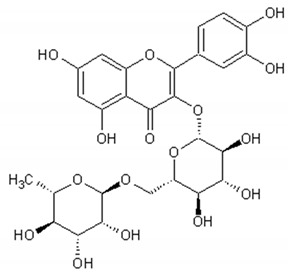	Antioxidant, cytoprotective, anti-inflammatory, immunomodulator, neuroprotective, neurodegenerative diseases protector, antitumor, antidiabetic, hypotensor, hyperlipidemia protector, antiviral	[131,132,133,134]
Apigenin	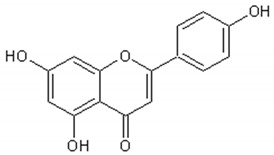	Antiinflammatory, antimicrobial, antitumor, antioxidant	[135,136,137,138,139]
Isorhamnetin	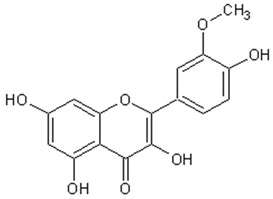	Antiinflammatory, antitumor, antioxidant, neuroprotective, antidiabetic	[140,141,142,143,144,145]
Kaempferol	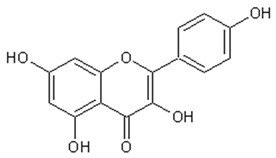	Antiinflammatory, antioxidant, neuroprotective, antitumor	[146,147,148,149]
Mearnsetin	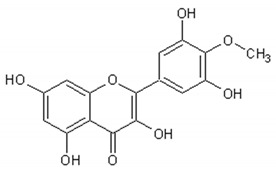	Antioxidant	[150]
Artemetin	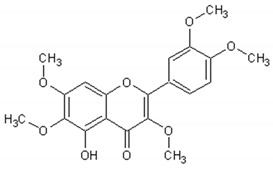	Hypotensor, antitumor, antioxidant, antiinflammatory	[151,152,153,154,155]
Casticin	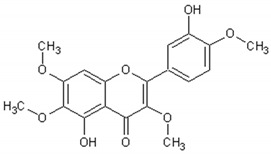	Antitumor, antiinflammatory, antioxidant, antiaging	[155,156,157,158]
Chrysosplenetin	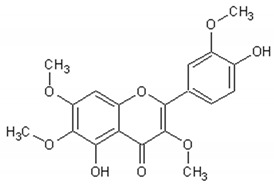	Antiviral	[159]
Chrysoprenol D	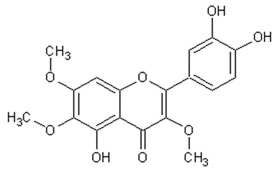	Antiinflammatory, antitumor, antioxidant	[33,95,160]
Cirsilineol	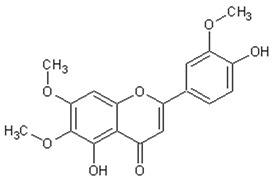	Immunosuppressive, antitumor	[161,162,163]
Eupatorine	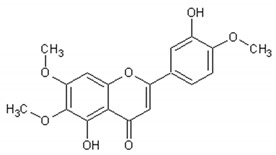	Antitumor	[164,165,166]

**Table 4:** Structure and biological activities of major coumarins of *Artemisia annua*.

Molecule	Structure	Activities	Ref.
Scopolin	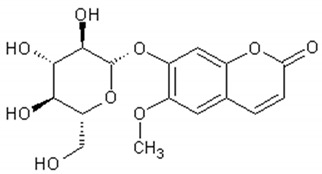	Antinociceptive, antiinflammatory, antioxidant, antipyretic, cooling effect, antiallergic	[167,168,169,170,171,172]
Scopoletin	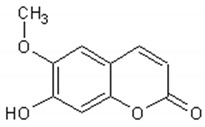		

## Abbreviations

ABTS	Acide 2,2′-azino-bis(3-éthylbenzothiazoline-6-sulphonique)
ACP	Acidic Phosphatases
ART	Artesunate
AD	Alzheimer’s Disease
ALDO	Aldolase
ALP	Alkaline Phosphatases
BAFF	B cell-activating factor
BKV	Polyomavirus BK
BVDV	Bovine Viral Diarrhea Virus
CAM	Chorioallantoic Membrane
CMV	CytoMegaloVirus
COX-2	Cyclooxygenase 2
CytC	Cytochrome C peroxidase
DAPI	Phenylindole dihydrochloride
DENV	Dengue Virus
DHA	Dihydroartemisinin
DPPH	2,2-diphényl-1-picrylhydrazyle
DSS	Sulfate Sodium Salt
EBV	Epstein-Barr Virus
ENO	Enolase
GAPDH	Glyceraldehyde-3-Phosphate Dehydrogenase
G6PD	Glucose-6-Phosphate Dehydrogenase
GPI	Glucose-6-Phosphate Isomerase
GPx	Glutathione Peroxidase
GR	Glutathione Reductase
GRα	Glucocorticoid receptor α
GSH	Glutathione
GST	Glutathione S-Transferase
HBsAg	HBV surface antigen
HBV	Hepatitis B Virus
HCMV	Human Cytomegalovirus
HCV	Hepatitis C Virus
HHC-6A	Human Herpes Virus 6A
HIF-1α	Hypoxia-Inducible Factor-1α
HK	Hexokinase
HPV	Human Papillomavirus
HSV-1	Herpes Simplex Virus 1
IBD	Inflammatory Bowel Disease
IL-1β	Interleukin 1 beta
iNOS	Inducible Nitric Oxide Synthase
IP3	Inositol trisphosphate
JCPyV	Human JC Polyomavirus
LDH	Lactate Dehydrogenase
LDL	Low Density Lipoprotein
LPS	Lipopolysaccharide
MAPK	Mitogen Activated Protein Kinase
MCP-1	Monocyte Chemoattractant Protein 1
MDH	Malate Dehydrogenase
ME	Malic Enzyme
MMP-2	Metalloproteinase-2
MPI	Mannose-6-Phosphateisomerase
NF-κB	Nuclear Factor κB
Nrf-2	Nuclear Factor (erythroid-derived 2)-like 2
ORAC	Oxygen Radical Absorbance Capacity
PARP	Poly (ADP-ribose) Polymerase
PFK	Phosphofructokinase
PGAM	Phosphoglycerate Mutase
PGD	6-Phosphogluconate Dehydrogenase
PGK	Phosphoglycerate Kinase
PI3K	Phosphoinositide 3-kinase
PK	Pyruvate Kinase
PLCγ	Phospholipase Cγ
RA	Rheumatoid Arthritis
ROS	Reactive Oxygen Species
SLE	Systemic Lupus Erythematosus
SM905	1-(12β-dihydroartemisinoxy)-2-hydroxy-3-tert-butylaminopropane maleate, new water-soluble derivative
SM934	β-aminoarteether maleate, new water-soluble derivative
SOD	Superoxide Dismutase
TGF-1β	Transforming Growth Factor beta 1
TNBC	Triple Negative Breast cancer
TNBS	Trinitrobenzene Sulfonic acid
TNF-α	Tumor Necrosis Factor alpha
VEGF	Vascular Endothelial Growth Factor
